# Comparación entre pruebas treponémicas y no treponémicas para la detección de sífilis

**DOI:** 10.7705/biomedica.7453

**Published:** 2025-09-22

**Authors:** Verónica Aguaiza, Rosa F. Chiriboga-Ponce

**Affiliations:** 1 Centro de Investigación para la Salud en América Latina (CISeAL), Pontificia Universidad Católica del Ecuador, Quito, Ecuador Pontificia Universidad Católica del Ecuador Pontificia Universidad Católica del Ecuador Quito Ecuador; 2 Carrera de Laboratorio Clínico, Pontificia Universidad Católica del Ecuador, Quito, Ecuador Pontificia Universidad Católica del Ecuador Pontificia Universidad Católica del Ecuador Quito Ecuador

**Keywords:** Treponema, sífilis, técnicas y procedimientos diagnósticos, bancos de sangre, Treponema, syphilis, diagnostic techniques and procedures, blood banks

## Abstract

**Introducción.:**

Tradicionalmente, la sífilis se ha detectado con pruebas no treponémicas y se ha confirmado con pruebas treponémicas, un algoritmo diagnóstico implementado a nivel mundial.

**Objetivo.:**

Evaluar el desempeño de las pruebas treponémicas y no treponémicas para detectar en forma efectiva la sífilis entre los donantes de sangre.

**Materiales y métodos.:**

Se realizó un estudio transversal con 384 muestras, inicialmente reactivas para sífilis según los resultados de pruebas no treponémicas. Posteriormente, se reevaluaron todas las muestras mediante VDRL, ELISA, CLIA y FTA-ABS, esta última como prueba confirmatoria. Se calculó el coeficiente kappa para determinar la concordancia entre las pruebas no treponémicas y se estimaron los índices de desempeño.

**Resultados.:**

Se determinó la concordancia entre las pruebas no treponémicas y las treponémicas. Entre la VDRL y ELISA, fue del 78,8 %, mientras que, entre la VDRL y el CLIA, fue del 76 % (p < 0,005). La concordancia entre el ELISA y el CLIA fue del 83 %. Al comparar los resultados obtenidos en las pruebas treponémicas y las no treponémicas con la FTA-ABS, la concordancia osciló entre el 44,2 y el 61,9 %. Los índices de desempeño evidenciaron que las pruebas no treponémicas y las treponémicas tienen valores de sensibilidad entre el 89,70 y el 99,39 %. El valor predictivo positivo fue más elevado en las pruebas treponémicas (CLIA = 95,27 %) y la tasa más alta de falsos positivos fue del 94,52 % en la prueba VDRL.

**Conclusiones.:**

Las muestras analizadas fueron reactivas desde el inicio, lo que podría interferir en los parámetros de medición. Sin embargo, aportaron información valiosa para evaluar los algoritmos implementados.

## Abstract (en)

**Introduction.:**

Syphilis detection has traditionally relied on non-treponemal tests for screening followed by confirmation with a treponemal test, an algorithm implemented worldwide.

**Objective.:**

To evaluate the performance of treponemal and non-treponemal tests in the effective detection of syphilis among blood donors.

**Materials and methods.:**

A cross-sectional study was conducted with 384 samples, initially reactive for syphilis by non-treponemal tests (VDRL). All samples were retested by VDRL, ELISA, CLIA, and FTA-ABS, the latter as a confirmatory test. We calculated kappa coefficients to establish agreement between tests and determine performance indices for each assay.

**Results.:**

We identified agreement between non-treponemal and treponemal tests. Concordance between VDRL and ELISA was 78.8% and between CLIA and VDRL was of 76% (p < 0.005). The concordance between ELISA and CLIA was 83%. When comparing the results of treponemal and non-treponemal tests with FTA-ABS, concordance rates ranged from 44.2% to 61.9%. Performance indices showed that non-treponemal and treponemal tests exhibit sensitivity values between 89.70% and 99.39%. The positive predictive value was higher for the CLIA test (95.27%), whereas the highest false-positive rate was observed for the VDRL test (94.52%).

**Conclusions.:**

The samples analyzed were reactive from the outset, which could interfere with the measurement parameters. However, they provided valuable information to evaluate the implemented algorithms.

La transfusión sanguínea, aunque es beneficiosa y salva vidas, conlleva riesgos, entre ellos, la transmisión de enfermedades infecciosas como la sífilis. A pesar de que la prueba para esta infección fue uno de los primeros tamizajes obligatorios a nivel mundial ^1^, se han reportado casos de transmisión posteriores a una transfusión.

Una de las causas puede ser la elección de las pruebas de detección, clasificadas como no treponémicas y treponémicas en función del antígeno diana de la prueba (con derivado de *Treponema pallidum* o sin él) ^2^. Las pruebas no treponémicas son inespecíficas; contienen anticuerpos antifosfolipídicos que detectan antígenos que se derivan de las mitocondrias de las células huésped como resultado de la destrucción del tejido provocada por la bacteria ^1^^,^^2^. Por su parte, las pruebas treponémicas detectan anticuerpos contra antígenos específicos de *T. pallidum*^1^^,^^2^.

Un aspecto que podría generar fallos en el desempeño de la prueba es la etapa de la enfermedad que esté cursando el donante o paciente, así como su reacción inmunológica. Los títulos de anticuerpos reagínicos o inespecíficos contra *T. pallidum* disminuyen en la segunda fase de la infección y pueden ser indetectables en personas tratadas o no tratadas. Por el contrario, en personas que tuvieron la enfermedad, hayan sido tratadas o no, los anticuerpos son detectados al utilizar pruebas treponémicas ^1^. Las pruebas no treponémicas (sin automatización) más comúnmente utilizadas, son la del *Venereal Disease Research Laboratory* (VDRL) y la de reagina plasmática rápida (RPR) ^2^, mientras que las pruebas treponémicas más aplicadas son el ensayo de inmunoabsorción ligado a enzimas (ELISA) y el inmunoensayo quimioluminiscente (CLIA) o electroquimioluminiscente (ECLIA).

En Ecuador, los bancos de sangre han migrado de usar pruebas no treponémicas a usar las treponémicas, acatando la directriz de la Organización Mundial de la Salud (OMS) que recomienda incluir pruebas sensibles y específicas para evitar la transmisión bacteriana.

Con base en lo anterior, el presente estudio tuvo como objetivo comparar el desempeño analítico de las pruebas utilizadas para la detección de sífilis en donantes de sangre, como aporte a la inclusión o mantenimiento del algoritmo en bancos de sangre.

## Materiales y métodos

Se recolectaron 384 muestras reactivas para VDRL durante seis meses en el 2019. Estas muestras se evaluaron mediante procedimientos de rutina utilizados en los bancos de sangre ecuatorianos: pruebas no treponémicas del *Venerai Disease Research Laboratory* (VDRL-I y VDRL-II), provenientes de dos casas comerciales diferentes, y pruebas treponémicas como el ELISA para detectar anticuerpos IgM e IgG contra TpN15, TpN17 y TpN47, y el CLIA para el reconocimiento de los mismos antígenos.

La prueba de absorción con anticuerpos treponémicos fluorescentes (FTA-ABS) se empleó como método de confirmación. En cada estudio se utilizaron controles de calidad internos. Para determinar la concordancia de los resultados de las pruebas no treponémicas, se realizó la comparación entre operadores mediante el coeficiente kappa.

### Análisis estadístico

Se utilizó estadística descriptiva para establecer frecuencias y porcentajes. Para determinar la relación entre los resultados obtenidos en pruebas serológicas (VDRL) y treponémicas (ELISA-CLIA), se utilizó la prueba de ji al cuadrado. Finalmente, se calcularon los parámetros de sensibilidad, especificidad, valores predictivos positivo y negativo, razón de probabilidades, y tasas de falsos positivos y negativos.

### Consideraciones éticas

Todos los donantes firmaron el consentimiento informado y el documento MSP-DNEAIS-PNS-FORM.22-ADSME de donantes de sangre antes de la toma de la muestra. Los investigadores no tuvieron contacto directo con los donantes de sangre y las muestras se recodificaron para garantizar el anonimato de los datos personales antes de su análisis.

## Resultados

Del total de muestras analizadas, el 91,4 % (351/384) de los donantes de sangre fueron compensatorios y únicamente el 8,6 % (33/384) fueron voluntarios. El promedio de edad de los donantes fue de 40 años (rango entre 18 y 65), con predominio del 69,5 % del sexo masculino (267/384).

En las pruebas no treponémicas se obtuvo un 75 % (288/384) de reactividad con la prueba denominada VDRL-I, mientras que, con la segunda - la VDRL-II-, se obtuvo un 96,6 % (371/384). El coeficiente kappa entre los dos estudios fue 0,98. En cuanto a las pruebas treponémicas, con ELISA se identificó el 73,4 % (282/384) de las muestras reactivas, mientras que, con el CLIA, se detectó el 66,9 % (257/384).

Respecto a la correlación entre las pruebas no treponémicas y las treponémicas, se encontró una concordancia del 78,8 % (227/288) entre las muestras reactivas con VDRL-I y ELISA, mientras que, entre las muestras no reactivas con las dos pruebas, fue del 42,7 % (41/96). La comparación entre VDRL-I y ELISA resultó estadísticamente significativa (p < 0,005). Por otra parte, al contrastar VDRL-II y ELISA, se encontró una correlación del 73,6 % (273/371) entre las muestras reactivas y del 33,3 % (4/12) entre las no reactivas. Estas diferencias no fueron estadísticamente significativas (p > 0,005) (cuadro 1). Al comparar la VDRL-I y el CLIA, se encontró una concordancia entre las muestras reactivas del 76 % (219/288).

**Cuadro 1:** Correlación entre las pruebas treponémicas y las no treponémicas

ELISA (IgG TOTAL)				
		No reactivas	Reactivas	p
		n %	n %	
VDRL-I	No reactivas	41 42,7	55 57,3	0,001
	Reactivas	61 21,2	227 78,8	
	No reactivas	4 33,30	9 66,70	0,723
VDRL-II	Reactivas	98 26,4	273 73,60	
CLIA				
		No reactivas	Reactivas	p
		n %	n %	
VDRL-I	No reactivas	58 60,4	38 39,6	0,001
	Reactivas	69 24,0	219 76	
	No reactivas	8 66,70	4 33,30	0,015
VDRL-II	Reactivas	118 31,80	253 68,20	

NR: no reactivo; R: reactivo; CLIA: *Chemiluminescent Immunoassay;* ELISA: *Enzyme-Linked Immunosorbent Assay* VDRL: *Venereal Disease Research Laboratory*

La concordancia entre las pruebas treponémicas ELISA y CLIA fue del 82,3 % (234/284) en las muestras reactivas y del 22,5 % (28/102) en las muestras no reactivas (p < 0,005) (cuadro 2).

**Cuadro 2:** Correlación entre las pruebas treponémicas

CLIA						
		No reactivas		Reactivas		p
		n	%	n	%	
ELISA	No reactivas	79	77,5	23	22,5	0,001
	Reactivas	48	17,0	234	83,0	

CLIA: *Chemiluminescent Immunoassay;* ELISA: *Enzyme-Linked Immunosorbent Assay*

Al comparar los resultados de las pruebas treponémicas y no treponémicas con la FTA-ABS, se encontró una correlación entre el 83,3 y el 95,3 % en las muestras no reactivas, mientras que, en las reactivas, el porcentaje disminuyó a un rango entre el 44,2 y el 61,9 %. Estas diferencias fueron estadísticamente significativas (p < 0,005) (cuadro 3).

El cálculo de los índices de desempeño mostró que las pruebas no treponémicas y las treponémicas tienen valores de sensibilidad entre el 89,70 y el 99,39 %. En cuanto a la especificidad, se detectó que la prueba no treponémica VDRL-II tuvo el valor más bajo (5,48 %), mientras que la prueba treponémica CLIA obtuvo el valor más alto (55,25 %). El valor predictivo positivo de las pruebas no treponémicas fue más alto para VDRL-I (51,38 %) y, en las pruebas treponémicas, fue más alto para CLIA (95,27 %); el valor predictivo negativo en las pruebas no treponémicas fue mayor para VDRL-II (94,52 %) y, en las treponémicas, fue mayor para CLIA (95,27 %).

Finalmente, la tasa de falsos positivos para VDRL-II fue la más alta (94,52 %) mientras que, en las pruebas treponémicas, ELISA obtuvo el valor más bajo (61,18 %); al determinar la tasa de falsos negativos, las pruebas VDRL-I (10,30 %) y ELISA (9,70 %) tuvieron los valores más altos, mientras que VDRL-II (3,63 %) y CLIA (0,60 %) mostraron los más bajos (cuadro 4).

**Cuadro 3:** Concordancia entre los resultados de la FTA-ABS, y las pruebas treponémicas y las no treponémicas

CLIA						
		No reactivas		Reactivas		p
		n	%	n	%	
ELISA	Reactivas	148	52,5	134	47,5	0,001
	No reactivas	17	16,7	85	83,3	
CLIA	Reactivas	159	61,9	98	38,1	0,001
	No reactivas	6	4,7	121	95,3	
VDRL-I	Reactivas	148	51,4	140	48,6	0,001
	No reactivas	17	17,7	79	82,3	
VDRL-II	Reactivas	164	44,2	207	55,8	0,032
	No reactivas	1	8,3	12	91,7	

FTA-ABS: *Fluorescent Treponemal Antibody-Absorption test;* CLIA: *Chemiluminescent Immunoassay;* ELISA: *Enzyme-Linked Immunosorbent Assay;* VDRL: *Venereal Disease Research Laboratory*

**Cuadro 4:** Índices de desempeño

	Sensibilidad	Especificidad	Valor predictivo positivo	Valor predictivo negativo	Tasa de falsos positivos	Tasa de falsos negativos
Fórmula	VP/VP + FN	VN / VN + FP	VP/VP + FP	VN/VN + FN	FP / (VN + FP)	FN / (VP + FN)
VDRL-I	89,70	36,07	51,38	82,29	64,00	10,30
VDRL-II	99,39	5,48	44,20	92,30	94,52	0,60
ELISA	91,10	38,81	52,48	83,33	61,18	9,70
CLIA	96,36	55,25	61,86	95,27	44,74	3,63

VP: valor predictivo positivo; VN: valor predictivo negativo; FN: falsos negativos; FP: falsos positivos; VDRL: *Venereal Disease Research Laboratory;* CLIA: *Chemiluminescent Immunoassay;* ELISA: *Enzyme-Linked Immunosorbent Assay*

## Discusión

Los resultados discordantes entre las pruebas treponémicas y las no treponémicas, generalmente son resueltos mediante pruebas confirmatorias ^3^. Sin embargo, resulta necesario establecer un algoritmo de detección, especialmente en el contexto de los bancos de sangre. Actualmente, se manejan dos algoritmos: el tradicional y el de serología de secuencia inversa, utilizados en algunos laboratorios de América y Europa ^4^. Tong *et al.* determinaron una sensibilidad del 75,81 % al utilizar el algoritmo tradicional ^5^, valor inferior al observado en este estudio, el cual osciló entre el 89,70 y el 96,36 %. No obstante, se debe considerar que el criterio de inclusión en el presente estudio -como en otras investigaciones ^6^- fue la serología reactiva, lo que podría sesgar la estimación del parámetro de sensibilidad o reflejar la subjetividad del operador. Aun así, el coeficiente kappa fue de 0,98, lo que indica una buena correlación entre los resultados de las pruebas no treponémicas.

Se encontraron discrepancias en la reactividad de las muestras evaluadas mediante los dos tipos de pruebas VDRL (no treponémicas). A pesar de ello, en los algoritmos tradicionales, se continúa utilizando estas pruebas como método inicial para detectar la sífilis ^7^. Por su parte, los Centros para el Control y la Prevención de Enfermedades (CDC) han recomendado la detección serológica de *T. pallidum* mediante pruebas no treponémicas -VDRL- para detectar infecciones no tratadas ^8^, siempre que sea confirmada con pruebas treponémicas como ELISA y CLIA que, si bien son específicas, no pueden discriminar entre sífilis activa y previa ^9^^,^^10^. En diversos estudios se señala que, al utilizar pruebas treponémicas para el diagnóstico inicial, se puede subestimar el número de pacientes con sífilis temprana ^6^. Por otra parte, en poblaciones con alto riesgo, se han observado tasas elevadas de reactividad asociadas con falsos positivos. En el presente estudio, se determinó una tasa de falsos positivos del 61,18 % para el ELISA y del 44,74 % para el CLIA.

La concordancia observada entre las muestras que resultaron reactivas en las pruebas treponémicas y las no treponémicas fue del 78,8 %, mientras que, en las no reactivas, fue del 42,7 %. Attie *et al.* demostraron que el 3 % de los donantes que cursaban la fase aguda de la infección obtuvieron un resultado negativo en la prueba de VDRL, lo que confirma que estas pruebas no son fiables para utilizarse en los bancos de sangre ^2^. En contraste, Nayak *et al.* señalan que las pruebas serológicas no treponémicas presentan una gran reactividad en la etapa secundaria de la sífilis y una sensibilidad aumentada. Sin embargo, 1-2 % de los casos pueden ser falsos negativos debido probablemente al efecto prozona ^9^. Por el contrario, en la etapa de latencia, la reactividad de las pruebas no treponémicas disminuye. En este estudio, la prueba VDRL-I tuvo una tasa de falsos negativos del 10,30 %, pero este hallazgo no se correlacionó con la etapa de la enfermedad en la que se encontraban los donantes, lo que constituye un factor limitante del estudio.

La concordancia determinada entre las pruebas treponémicas, el ELISA y el CLIA, fue del 83 % en las muestras reactivas y del 22,5 % en las muestras no reactivas (p < 0,005). Estas pruebas treponémicas son importantes en el diagnóstico de la sífilis y han demostrado una gran sensibilidad y especificidad ^10^. Con la prueba treponémica CLIA, se obtuvo una sensibilidad del 96,36 %, mayor de la detectada por Jaramillo *et al.,* quienes reportaron un valor del 93,9 %. Sin embargo, este valor puede cambiar según el estadio de la enfermedad. En población con bajo riesgo riesgo, pueden aumentar los falsos positivos ^11^, como ocurrió en este estudio, en el cual fueron del 44,74 % para CLIA. Sin embargo, CLIA es un estudio de detección que está por fuera del algoritmo recomendado por los CDC de Estados Unidos, como lo reportaron Peng *et al.*^12^, lo que podría influir en la tasa de falsos positivos y en la interpretación de los resultados.

La eficacia de los métodos treponémicos que utilizan TpN17 y TpN47 es consistente. Silva *et al.* sugieren que el uso de estos antígenos en las pruebas treponémicas es independiente del área geográfica y que la variabilidad genética no influye en las pruebas diagnósticas ^13^. Aunque las pruebas treponémicas utilizadas en este estudio contienen los antígenos TpN15, TpN47 y TPN17, muestran una sensibilidad y especificidad variables, tal vez sesgadas por usarse solamente muestras reactivas. Este resultado es similar al del estudio de Serhir *et al.,* quienes utilizaron únicamente muestras con reactividad débil en las pruebas no treponémicas. Estas muestras resultaron reactivas en con el CLIA y se confirmaron con una segunda prueba treponémica (prueba de aglutinación de partículas para *Treponema pallidum,* TPPA), lo que evidenció una tasa de confirmación de sífilis del 100 %. Según estos resultados, los autores sugirieron que este algoritmo podría optimizar el serodiagnóstico de sífilis ^14^. Sin embargo, en varios estudios se ha evaluado la concordancia entre las pruebas treponémicas y las no treponémicas, y se ha concluido que siempre debe incluirse una prueba confirmatoria para evitar falsos positivos ^15^.

En el presente estudio, se utilizó la prueba FTA-ABS como método confirmatorio. Con estos resultados, se calculó que la sensibilidad de las pruebas treponémicas y las no treponémicas osciló entre el 89,70 y el 99,39 %, con una tasa de falsos positivos entre el 44 y el 94 %. En el estudio realizado por Li *et al.,* se utilizaron la TPPA como segunda prueba confirmatoria y se encontró un alto grado de concordancia entre las pruebas treponémicas (CLIA, ELISA y TPPA). En contraste, en el presente estudio, se observó el caso contrario, lo que resalta la importancia de considerar la historia de la enfermedad, así como los algoritmos diagnósticos utilizados. Esta situación constituye otra limitación en el presente estudio, ya que el banco de sangre no hace seguimiento de los donantes con resultados reactivos para sífilis.

El valor predictivo negativo fue elevado en todas las pruebas no treponémicas y las treponémicas, oscilando entre el 82,29 y el 95,27%. Attie *et al.* enfatizaron en su estudio que las pruebas no treponémicas no son las mejores para el cribado de donantes de sangre, debido a la subjetividad en la interpretación de los resultados.

Una fortaleza del presente estudio es que el tamizaje previo de las muestras incluidas en el análisis fue realizado por personal del banco de sangre siguiendo el protocolo tradicional, lo que facilita la implementación de un algoritmo adaptado a las características de la población que dona sangre. En la investigación de Binnicker *et al.,* se asegura que el algoritmo inverso identifica un mayor porcentaje de pacientes con sífilis -confirmada mediante TPPA o FTA-ABS- aunque muchos de los individuos positivos tuvieran sífilis tratadas o pasadas. Sin embargo, se enfatizó que las pruebas treponémicas (ELISA y CLIA) facilitan la detección de la sífilis en etapas latentes y tempranas, por lo que se recomienda su implementación ^16^. Sangthang *et al.* concluyeron que el uso del algoritmo inverso podría incrementar la tasa de descarte de hemocomponentes, pero aumentaría la seguridad transfusional. Asimismo, destacaron que la aplicación de este algoritmo puede variar según las estrategias diagnósticas y económicas de cada país ^17^.

Finalmente, la evaluación de los índices de desempeño de las pruebas utilizadas para detectar la sífilis, es necesaria para definir e implementar algoritmos eficaces en el tamizaje de donantes en los bancos de sangre. No obstante, deben considerarse factores como la prevalencia de la enfermedad, el estadio clínico y su evolución, aspectos de difícil seguimiento por parte del banco de sangre. Los estudios muestran que las tasas de detección y prevalencia de la sífilis van a estar influenciadas por las pruebas utilizadas durante el cribado de donantes de sangre.
